# Oxygen use and survival in patients with advanced cancer and low oxygen saturation in home care: a preliminary retrospective cohort study

**DOI:** 10.1186/s12904-019-0511-9

**Published:** 2020-01-03

**Authors:** Hiroshi Igarashi, Motoharu Fukushi, Naoki Nago

**Affiliations:** Musashi Kokubunji Park Clinic, 2-16-34-127 Nishimoto-machi, Kokubunji, Tokyo 185-0023 Japan

**Keywords:** Cancer, Dyspnea, Hypoxia, Oxygen, Prognosis, Survival

## Abstract

**Background:**

The role of oxygen therapy in end-of-life care for patients with advanced cancer is incompletely understood. We aimed to evaluate the association between oxygen use and survival in patients with advanced cancer and low oxygen saturation in home care.

**Methods:**

We conducted a retrospective cohort study at a primary care practice in suburban Tokyo. Adult patients in home care with advanced cancer demonstrating first low oxygen saturation (less than 90%) detected in home visits were consecutively included in the study. Cox proportional hazards regression was used to investigate the effect of oxygen use on overall survival and survival at home, adjusted for systolic blood pressure, decreased level of consciousness, dyspnea, oral intake, performance status, and cardiopulmonary comorbidity.

**Results:**

Of 433 identified patients with advanced cancer, we enrolled 137 patients (oxygen use, *n* = 35; no oxygen use, *n* = 102) who developed low oxygen saturation. In multivariable analysis, the adjusted hazard ratio (HR) of oxygen use was 0.68 (95% confidence interval 0.39–1.17) for death and 0.70 (0.38–1.27) for death at home. In patients with dyspnea, the HR was 0.35 (0.13–0.89) for death and 0.33 (0.11–0.96) for death at home; without dyspnea, it was 1.03 (0.49–2.17) for death and 0.84 (0.36–1.96) for death at home.

**Conclusions:**

Oxygen use was not significantly associated with survival in patients with advanced cancer and low oxygen saturation, after adjusting for potential confounders. It may not be necessary to use oxygen for prolongation of survival in such patients, particularly in those without dyspnea.

## Background

Low oxygen saturation is a common finding in patients with terminal cancer [1–3] and thus supplemental oxygen is frequently provided to patients with terminal cancer [4]. The use of oxygen is sometimes intended to alleviate dyspnea in patients with terminal cancer. However, it is not uncommon that the family hopes for prolonged survival, or healthcare providers recommend that oxygen be used for fear that not using it hastens death, especially in patients with low oxygen saturation. Studies have found that patients, caregivers, and healthcare providers perceive oxygen as life-sustaining [5–7]. The potential influence of oxygen use on survival in patients with terminal cancer and low oxygen saturation may have important implications for end-of-life decision-making.

The effect of oxygen on dyspnea in patients who are terminally ill has not been established. Randomized controlled trials have shown that oxygen, compared with air, was not effective in alleviating dyspnea in these patients in the absence of severe hypoxemia; that is, partial pressure of oxygen in arterial blood (PaO_2_) > 55–60 mmHg or oxygen saturation > 88–90% [8–11]. The use of oxygen for the relief of dyspnea in patients with advanced cancer who have low oxygen saturation is controversial [12–15].

Low oxygen saturation has been reported to be a risk factor for death in patients with advanced cancer [1–3, 16–18]. Randomized controlled trials have shown that long-term oxygen for patients with chronic obstructive pulmonary disease with severe hypoxemia resulted in reduced mortality [19, 20]. However, long-term oxygen provided no significant mortality benefit in patients with chronic obstructive pulmonary disease and resting oxygen saturation of 89–93%, or exercise-induced moderate desaturation [21]. In contrast, no randomized controlled trial has been conducted on the effect of oxygen in patients who have advanced cancer and low oxygen saturation. Observational studies have found that oxygen use was a risk factor for death [2, 16, 22, 23]. These results, however, may be confounded by low oxygen saturation before oxygen use. In addition, it is not clear that oxygen was used for patients with low oxygen saturation. Therefore, little is known about the association of oxygen use with survival in patients with advanced cancer who have low oxygen saturation.

Our study aimed to evaluate the association of oxygen use with survival in patients with advanced cancer demonstrating low oxygen saturation in home care, thereby informing end-of-life decision-making.

## Methods

### Study design and population

This was a cohort study conducted at a primary care practice in suburban Tokyo. Adult patients aged 20 years or older with locally advanced or metastatic cancer followed by a home medical care service provided by the practice were screened. Patients with first low oxygen saturation (peripheral arterial oxygen saturation measured by pulse oximetry < 90%) detected in home visits between June 1, 2011 and November 30, 2018 were retrospectively identified by chart review and consecutively included in the study. Oxygen saturation was measured by the physicians who visited the patient’s home or by the nurses who accompanied them, as part of the routine clinical practice for every patient under the home medical care service. All physicians and nurses measured oxygen saturation after ensuring that the patient was satisfactorily rested. Patients who had been on oxygen at the time of admission to the service and those for whom oxygen therapy was initiated before identification of decreased oxygen saturation were excluded from the study. The number of patients in the practice during the study period determined the sample size. Follow-up ended on November 30, 2018.

### Study variables

All data on study variables were obtained by retrospective chart review. The exposure was the use of oxygen at home. The decision regarding whether to start supplemental oxygen was made following discussion between the patient, the family and the physician. The patient and family’s wishes were respected. Whether to recommend supplemental oxygen was at the discretion of the attending physician, who did not always recommend oxygen to patients who developed low oxygen saturation. Oxygen was administered via a concentrator through a nasal cannula at 1–4 L/min (mean, 1.9 L/min). Administration of oxygen by a mask was allowed in case of nasal irritation. Flow rate was determined at the discretion of the physician and adjusted to maintain an oxygen saturation of 90% or more. Oxygen was administered continuously at a steady flow rate unless the physician advised changing it. Intermittent use or increase of flow rate on exertion within a range of 1–4 L/min was allowed at the patient’s request.

Major potential confounders (decreased level of consciousness, dyspnea, oral intake, and performance status) were selected a priori based on previous literature regarding prognostic factors for survival in patients with advanced cancer [24–26]. Other factors considered to be potential confounders were age, sex, systolic blood pressure [23], heart rate [16, 22, 23, 25–27], oxygen saturation [16–18], edema [24, 28], cardiopulmonary comorbidity (heart failure or chronic lung disease), and lung cancer as the primary tumor [25]. We considered demographics and factors that might affect survival in patients with advanced cancer based on previous literature or on clinical basis, to be potential confounders. Respiratory rate was excluded from the potential confounders considered because of large numbers of missing data. All of the potential confounders were assessed at the time of development of low oxygen saturation. We used Doctor Bayes (Macros Japan, Tokyo, Japan), a Bayes theorem–based clinical decision support system that can be used in conjunction with electronic health records for recording patients’ symptoms [29]. This system can record all patients’ reason-for-encounter and diagnosis codes. At each home visit, each patient’s symptoms were entered into Doctor Bayes, according to the International Classification of Primary Care, second edition (ICPC-2) codes, by one of the family physicians in the practice [30]. When patients were unable to self-report their symptoms, reports from their family were included in patients’ symptoms. Decreased level of consciousness, dyspnea, and edema were considered present when the ICPC-2 codes A07 (coma), R02 (shortness of breath/dyspnea), and K07 (swollen ankles/edema), respectively, were recorded in the patient’s medical record. Patients with confusion, drowsiness, or delirium were also included, together with comatose patients, in the decreased level of consciousness cohort. When the relevant ICPC-2 code was not recorded but there was a clear description of the symptoms in the patient’s medical record, that symptom was considered present. Oral intake and performance status were determined according to the description in the patient’s medical record. Oral intake was categorized into normal (able to eat as much as the patient ate when he or she was in good health), reduced (able to eat, but less than the amount the patient ate when he or she was in good health), or impossible (not able to eat at all). Performance status was recorded using the Eastern Cooperative Oncology Group Performance Status (ECOG) [31]. Cardiopulmonary comorbidity was considered present in patients with heart failure or chronic lung disease that possibly caused low oxygen saturation. Dyspnea was prespecified as a potential effect modifier.

Patients were followed with home visits, typically once or twice plus as needed in a week. Oxygen saturation at baseline, the highest, and the lowest reading during the follow-up at home were recorded according to the use of oxygen.

The main outcome measures were overall survival and survival at home. Overall survival was defined as the time from the development of low oxygen saturation until death, including instances after the end of follow-up at home. The date of death after the end of follow-up at home was ascertained by the report from the hospital, including the palliative care unit, to which the patient was admitted or referred. When it was not available, overall survival was censored at the end of follow-up at home. Survival at home was defined as the time from the development of low oxygen saturation until death at home. Patients were censored at the end of follow-up at home (e.g., hospital admission) for survival at home. We selected survival at home as one of the main outcome measures because oxygen use after the end of follow-up at home could not be ascertained and could potentially influence overall survival.

### Statistical analysis

Medians with corresponding interquartile range (IQR) were calculated for overall survival and survival at home using the Kaplan–Meier survival function. The statistical significance of estimates from Kaplan–Meier survival curves for overall survival and survival at home were tested using the log-rank test. The statistical significance of the difference in the proportion of patients who died was assessed using Fisher’s exact test, as was the proportion of patients who died at home.

Cox proportional hazards regression was used to calculate hazard ratios (HR) and 95% confidence intervals (CIs) for overall survival and survival at home, adjusted for potential confounders. We incorporated a priori variables and variables remained statistically significant at a level of *p* <  0.10 in the univariate analysis, into the final multivariate model.

Missing data were imputed with multiple imputation by chained equations (MICE). We assumed data were missing at random. We performed imputation with linear regression for systolic blood pressure and heart rate; predictive mean matching (k-nearest neighbors, k = 3) for oxygen saturation; logistic regression for decreased level of consciousness and edema; and ordered logistic regression for oral intake and performance status. Twenty imputed data sets were generated, with the results combined using Rubin’s Rules.

Potential effect modification of oxygen use by dyspnea was investigated by stratification. Complete-case analysis was also performed.

A *p*-value < 0.05 was considered statistically significant. All statistical analyses were performed using Stata Statistical Software: Release 14 (StataCorp LP, College Station, TX, USA).

## Results

**Fig. 1:**
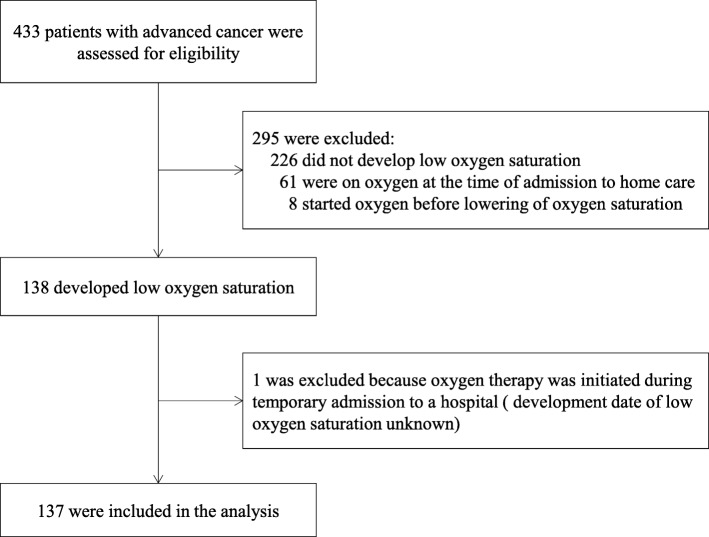
Selection of study participants

Of 433 adult patients with advanced cancer, a total of 137 patients (oxygen use, *n* = 35; no oxygen use, *n* = 102) who developed low oxygen saturation were consecutively included in the study (Fig. 1).

The baseline characteristics of the patients included in the study according to oxygen use are presented in Table 1. The median age of the patients was 77 years; 62% were men, and 85% were of ECOG Performance Status 3 or 4.

**Table 1:** Baseline characteristics of patients with advanced cancer according to oxygen use

	Oxygen use (*n* = 35)	No oxygen use (*n* = 102)	Total (*n* = 137)
Age, median (IQR), years	74 (69–80)	78 (69–83)	77 (69–82)
Sex, male, no. (%)	18 (51)	67 (66)	85 (62)
Systolic blood pressure, mean (SD), mmHg	117.0 (21.9)	104.1 (22.8)	107.3 (23.1)
Unknown, no. (%)	2 (5.7)	1 (1.0)	3 (2.2)
Heart rate, mean (SD), beats/min	102.7 (17.9)	94.1 (17.8)	96.3 (18.2)
Unknown, no. (%)	1 (2.9)	3 (2.9)	4 (2.9)
Respiratory rate, median (IQR), breaths/min	23 (18–33)	20 (16–27)	20 (16–30)
Unknown, no. (%)	13 (37)	45 (44)	58 (42)
Oxygen saturation, median (IQR), %	85 (82–87)	86 (83–88)	85 (83–88)
Unknown, no. (%)	1 (2.9)	0 (0)	1 (0.7)
Decreased level of consciousness, no. (%)	14 (40)	57 (56)	71 (52)
Unknown	0 (0)	1 (1.0)	1 (0.7)
Dyspnea, no. (%)	25 (71)	19 (19)	44 (32)
Unknown	0 (0)	0 (0)	0 (0)
Edema, no. (%)	13 (37)	48 (47)	61 (45)
Unknown	0 (0)	6 (5.9)	6 (4.4)
Oral intake, no. (%)			
Normal	8 (23)	10 (9.8)	43 (31)
Reduced	19 (54)	55 (54)	74 (54)
Impossible	7 (20)	36 (35)	18 (13)
Unknown	1 (2.9)	1 (1.0)	2 (1.5)
ECOG performance status, no. (%)			
1	0 (0)	1 (1.0)	1 (0.7)
2	9 (26)	8 (7.8)	17 (12)
3	17 (49)	46 (45)	63 (46)
4	9 (26)	44 (43)	53 (39)
Unknown	0 (0)	3 (2.9)	3 (2.2)
Cardiopulmonary comorbidity, no. (%)	11 (31)	4 (3.9)	15 (11)
Heart failure	5 (14)	3 (2.9)	8 (5.8)
Chronic obstructive pulmonary disease	4 (11)	1 (1.0)	5 (3.6)
Interstitial lung disease or radiation pneumonitis	4 (11)	1 (1.0)	5 (3.6)
Severe asthma	1 (2.9)	0 (0)	1 (0.7)
Primary tumor sites, no. (%)			
Gastrointestinal	11 (31)	53 (52)	64 (47)
Lung	14 (40)	23 (23)	37 (27)
Urologic	3 (8.6)	14 (14)	17 (12)
Hematologic	4 (11)	3 (2.9)	7 (5.1)
Head and neck	1 (2.9)	4 (3.9)	5 (3.6)
Unknown	2 (5.7)	2 (2.0)	4 (2.9)
Gynecologic	1 (2.9)	2 (2.0)	3 (2.2)
Breast	0 (0)	2 (2.0)	2 (1.5)
Central nervous system	1 (2.9)	1 (1.0)	2 (1.5)
Skin (melanoma)	0 (0)	1 (1.0)	1 (0.7)

*ECOG* Eastern Cooperative Oncology Group, *IQR* Interquartile range, *SD* Standard deviation

**Fig. 2:**
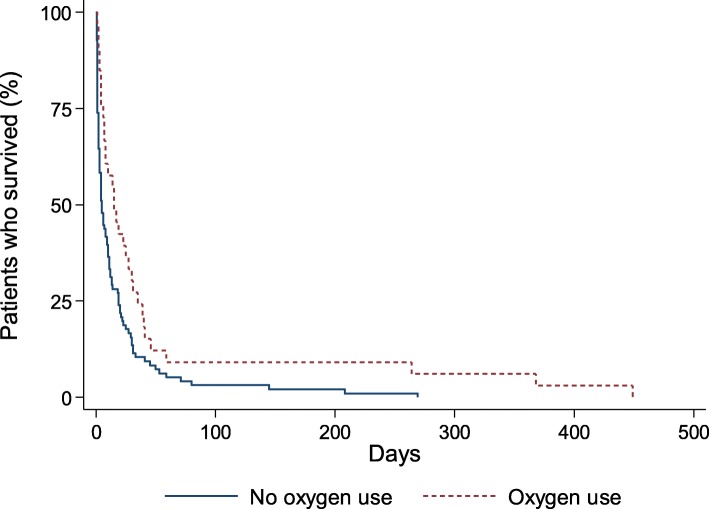
Kaplan-Meier curves of overall survival according to oxygen use

Outcome data of the patients included in the study according to oxygen use are presented in Table 2. Oxygen saturation during follow-up at home was higher in patients who used oxygen compared with those who did not. During a median of 8 days (IQR 2–23) (oxygen use, 15 days [IQR 6–34]; no oxygen use, 5 days [IQR 1–19]) of the entire follow-up, 129 patients (94%) died. During a median of 6 days (IQR 2–19) (oxygen use, 14 days [IQR 5–32]; no oxygen use, 4 days [IQR 1–32]) of follow-up at home, 100 patients (73%) died at home. There was no significant difference in the proportion of patients who died between the two groups (*p* = 1.0), nor in the proportion of those who died at home (*p* = 0.28). The median overall survival was 7 days (IQR 2–23) (oxygen use, 15 days [IQR 6–35]; no oxygen use, 5 days [IQR 1–19]; *p* = 0.008) (Fig. 2). The median survival at home was 10 days (IQR 2–31) (oxygen use, 19 days [IQR 7–46]; no oxygen use, 6 days [IQR 2–29]; *p* = 0.017).

**Table 2:** Patient outcome according to oxygen use

Oxygen saturation, median (IQR), %	Oxygen use (*n* = 35)	No oxygen use (*n* = 102)	Total (*n* = 137)
Oxygen saturation at baseline	85 (82–87)	86 (83–88)	85 (83–88)
Highest oxygen saturation during follow-up at home	96 (94–98)	89 (85–96)	93 (88–97)
Lowest oxygen saturation during follow-up at home	83 (80–86)	84 (80–87)	84 (80–87)
Time from the development of low oxygen saturation to the initiation of oxygen therapy, median (IQR), days	0 (0–4)	–	–
Outcome at end of entire follow-up, no. (%)	Oxygen use (*n* = 35)	No oxygen use (*n* = 102)	Total (*n* = 137)
Died	33 (94)^a^	96 (94)^a^	129 (94)
Admitted to a hospital including PCU	2 (5.7)	5 (4.9)	7 (5.1)
Switched to outpatient follow-up	0 (0)	1 (1.0)	1 (0.7)
Outcome at end of follow-up at home, no. (%)	Oxygen use (*n* = 35)	No oxygen use (*n* = 102)	Total (*n* = 137)
Died at home	23 (66)^b^	77 (75)^b^	100 (73)
Admitted to a hospital including PCU	12 (34)	23 (23)	35 (26)
Died during emergency transport	0 (0)	1 (1.0)	1 (0.7)
Switched to outpatient follow-up	0 (0)	1 (1.0)	1 (0.7)
Survival, median (IQR), days	Oxygen use (*n* = 33)	No oxygen use (*n* = 96)	Total (*n* = 129)
Overall survival	15 (6–35)	5 (1–19)	7 (2–23)
	(*n* = 35)	(*n* = 102)	(*n* = 137)
Survival at home	19 (7–46)	6 (2–29)	10 (2–31)

*IQR* Interquartile range, *PCU* Palliative care unit

^a^*p* = 1.0 for the difference in the proportion of patients who died (Fisher’s exact test)

^b^*p* = 0.28 for the difference in the proportion of patients who died at home (Fisher’s exact test)

Univariate analysis of oxygen use and potential confounders for survival are presented in Table 3. The unadjusted HR of oxygen use was 0.59 (95% CI 0.39–0.88; *p* = 0.011) for death and 0.58 (95% CI 0.36–0.92; *p* = 0.021) for death at home.

**Table 3:** Univariate analysis of oxygen use and potential confounders for survival

	Death		Death at home	
	Unadjusted HR (95% CI)	*p*-value	Unadjusted HR (95% CI)	*p*-value
Oxygen use	0.59 (0.39–0.88)	0.011	0.58 (0.36–0.92)	0.021
Age	0.99 (0.97–1.00)	0.16	0.99 (0.98–1.01)	0.50
Sex, male	1.33 (0.93–1.91)	0.12	1.25 (0.83–1.89)	0.28
Systolic blood pressure	0.99 (0.98–1.00)	0.008	0.98 (0.97–0.99)	0.002
Heart rate	1.00 (0.99–1.01)	0.94	1.01 (1.00–1.02)	0.24
Oxygen saturation	1.02 (0.98–1.06)	0.28	1.02 (0.98–1.07)	0.27
Decreased level of consciousness	2.26 (1.57–3.24)	< 0.001	2.81 (1.86–4.24)	< 0.001
Dyspnea	0.83 (0.57–1.20)	0.32	0.98 (0.64–1.49)	0.92
Edema	1.09 (0.76–1.55)	0.64	1.14 (0.76–1.71)	0.51
Oral intake	0.48 (0.36–0.62)	< 0.001	0.31 (0.22–0.43)	< 0.001
Performance status	1.68 (1.29–2.18)	< 0.001	2.14 (1.59–2.87)	< 0.001
Cardiopulmonary comorbidity	0.44 (0.25–0.80)	0.006	0.46 (0.23–0.91)	0.026
Lung cancer as primary tumor	0.76 (0.51–1.14)	0.18	0.73 (0.45–1.17)	0.19

*CI* Confidence interval, *HR* Hazard ratio

Multivariate analysis of oxygen use and potential confounders for survival are presented in Table 4. We incorporated a priori variables and variables remained statistically significant at a level of *p* <  0.10 in the univariate analysis, into the final multivariate model. Consequently, the multivariate model was adjusted for systolic blood pressure, decreased level of consciousness, dyspnea, oral intake, performance status, and cardiopulmonary comorbidity. The adjusted HR of oxygen use was 0.68 (95% CI 0.39–1.17; *p* = 0.16) for death and 0.70 (95% CI 0.38–1.27; *p* = 0.24) for death at home.

**Table 4:** Multivariate analysis of oxygen use and potential confounders for survival

	Death		Death at home	
	Adjusted HR (95% CI)	*p*-value	Adjusted HR (95% CI)	*p*-value
Oxygen use	0.68 (0.39–1.17)	0.16	0.70 (0.38–1.27)	0.24
Systolic blood pressure	1.00 (0.99–1.01)	0.41	1.00 (0.99–1.01)	0.75
Decreased level of consciousness	1.90 (1.21–2.99)	0.005	2.01 (1.22–3.33)	0.006
Dyspnea	1.22 (0.75–1.99)	0.43	1.25 (0.75–2.11)	0.39
Oral intake	0.55 (0.40–0.76)	< 0.001	0.37 (0.24–0.55)	< 0.001
Performance status	0.92 (0.64–1.32)	0.65	1.09 (0.74–1.61)	0.66
Cardiopulmonary comorbidity	0.90 (0.47–1.74)	0.75	1.04 (0.48–2.26)	0.92

*CI* Confidence interval, *HR* Hazard ratio

Effect modification of oxygen use by dyspnea is summarized in Table 5. The adjusted HR of oxygen use was 0.35 (95% CI 0.13–0.89; *p* = 0.027) for death and 0.33 (95% CI 0.11–0.96; *p* = 0.041) for death at home in patients with dyspnea. In contrast, the adjusted HR of oxygen use was 1.03 (95% CI 0.49–2.17; *p* = 0.94) for death and 0.84 (95% CI 0.36–1.96; *p* = 0.68) for death at home in patients without dyspnea.

**Table 5:** Effect modification of oxygen use by dyspnea

	Death			Death at home		
	No. (%)	Adjusted HR (95% CI)	*p*-value	No. (%)	Adjusted HR (95% CI)	*p*-value
Dyspnea present (*n* = 44)	42 (95)	0.35 (0.13–0.89)	0.027	32 (73)	0.33 (0.11–0.96)	0.041
Dyspnea absent (*n* = 93)	87 (94)	1.03 (0.49–2.17)	0.94	68 (73)	0.84 (0.36–1.96)	0.68

*CI* Confidence interval, *HR* Hazard ratio

Complete-case analysis yielded similar results as those in the analysis using multiple imputation (data not shown).

## Discussion

In this retrospective cohort study of patients with advanced cancer and low oxygen saturation in home care, oxygen use was not significantly associated with survival, after adjusting for known prognostic factors and cardiopulmonary comorbidity. Oxygen use was associated with longer survival in patients with dyspnea, whereas no significant association was found in patients without dyspnea.

To our knowledge, this study is the first to investigate the association of oxygen use with survival in patients with advanced cancer who have low oxygen saturation. As expected, in univariate analysis, survival was longer in patients who used oxygen compared with those who did not. In multivariate analysis, however, there was no significant difference in survival between the two groups, after adjusting for potential confounders. This study suggests that, while oxygen tended to be foregone in patients with serious illness who were expected to die within several days, oxygen use per se had no significant effect on survival. In this study, more patients who did not use oxygen had risk factors for shorter survival (i.e., decreased level of consciousness, decreased oral intake, and lower performance status) at baseline, compared with those who used oxygen. This finding is consistent with previous studies, in which a diagnosis of “terminal” or “dying” was associated with decisions about the limitation of treatment in patients with cancer [32, 33]. The result of this study seems plausible, both clinically and biologically, for several reasons. First, the patient population in this study had a relatively short survival, with a median overall survival of 7 days. Patients with such severe illness may have had a short duration of survival, whether they used oxygen or not. Our findings parallel the result of a randomized controlled trial, in which there was no significant difference in survival between the parenteral hydration and placebo groups in patients with advanced cancer and dehydration [34]. Second, a low oxygen saturation reading by pulse oximetry may not necessarily be an accurate reflection of arterial hypoxemia. Falsely low readings of oxygen saturation by pulse oximetry may occur in various settings and medical conditions, such as hypoperfusion [35, 36]. While pulse oximetry is thus limited, evaluating hypoxemia by arterial blood gas is invasive and may not be suitable in palliative care settings. Third, excessive oxygen supplementation may be harmful as was shown in a meta-analysis of randomized controlled trials, in which liberal oxygen therapy increased mortality in acutely ill adults [37].

Our findings suggest that, in patients with advanced cancer and low oxygen saturation, it may not be necessary to use oxygen for prolongation of survival, particularly in those without dyspnea. Although the exploratory nature of our study precludes definitive conclusion, we believe that the use of oxygen in patients without dyspnea cannot be recommended at present because it may interfere with daily activities, produce nasal irritation, and increase the cost of care. A randomized controlled trial or a larger prospective cohort study is needed to confirm our findings, including the possible prolongation of survival by oxygen use in patients with dyspnea. In such patients, the decision whether to use oxygen or not should be individualized, taking into consideration the uncertainty of its effect on dyspnea and patients’ and caregivers’ perceptions of oxygen therapy. Of note, fan therapy may be an effective and less expensive alternative for the relief of dyspnea in patients with advanced cancer [38–40].

This study has several limitations. First, we cannot entirely rule out the possibility of unmeasured or unknown confounders that may influence the results because this was an observational study. For example, clinical prediction of survival has been found to be a prognostic factor for survival in patients with advanced cancer, although it tended to overestimate the length of actual survival [25, 26, 41, 42]. It may not change our conclusions, however, because the bias it could have introduced would overestimate the effect of oxygen use on survival, provided that physicians were more likely to use oxygen in patients with longer life expectancy. Respiratory rate was another potential confounder that we could not adjust for because of a large amount of missing data. A few studies have found that respiratory rate was a prognostic factor for survival [27, 28]. Other factors that we could not adjust for were etiologies of dyspnea/ low oxygen saturation (parenchymal pulmonary involvement other than lung cancer as the primary tumor, including pneumonia and lymphangitis carcinomatosa); the acuity and persistence of desaturation; and concurrent pharmacological treatment of dyspnea other than supplemental oxygen, as well as palliative sedation. Etiologies of dyspnea/ low oxygen saturation could not be determined in most cases because of the limited diagnostic resources in home care. Although we ascertained the highest and lowest readings of oxygen saturation during the follow-up at home (Table 2), we could not assess the acuity and persistence of desaturation completely. As for concurrent use of medications for dyspnea, we did not consider them as potential confounders because previous literature suggested that opioid use at the end of life [43, 44] or palliative sedation [45–47] were not associated with patient survival. Although we adjusted for major prognostic factors for survival in patients with advanced cancer, the relatively small sample size of our study did not allow us to assess potential confounders comprehensively. Second, this study could be underpowered to detect the statistical significance for survival between the two groups because its sample size was relatively small. Based on this preliminary study, we calculated that 587 patients would be needed to detect a multivariate-adjusted hazard ratio of 0.68 for death with 90% power, a two-sided alpha level of 0.05 and 94% event rate of death. To meet the sample size requirement, we plan to continue recruiting participants to our study, the results of which will be reported in their entirety in the future. Third, we may not have identified all patients with low oxygen saturation, particularly in the last few days of their lives. The prevalence of low oxygen saturation in this study was lower than those in previous studies [1–3]. Those investigators measured oxygen saturation more frequently (e.g., twice daily) than we did in this study (only on the day we visited the patient’s home, typically once or twice plus as needed in a week). Fourth, the retrospective design of this study could have led to some misclassifications of potential confounders, particularly for oral intake and performance status, which we determined according to the description in the patient’s medical record. Fifth, although patients were checked for their symptoms including dyspnea and delirium on every visit, recording of these symptoms depended on the subjective report of the patient (or their family if the patient could not convey their symptoms) because no validated assessment tool was used for screening dyspnea or delirium. This could have led to under (or over)-estimation of these symptoms especially in patients who had difficulty in reporting their symptoms. Sixth, in terms of supplemental oxygen, intermittent use was allowed at the patient’s request. Patients may have applied intermittent use of oxygen frequently due to its inconvenience at the end of life. We do not have data regarding what proportion of patients used supplemental oxygen constantly or intermittently, which may have influenced the outcomes. Seventh, the pathophysiological basis of the effect modification of oxygen by dyspnea is unclear. Dyspnea was prespecified as a potential effect modifier because, if there was an effect modification by dyspnea, we presumed it to be clinically useful when considering the use of supplemental oxygen. Eighth, we could not measure dyspnea and quality of life as outcomes in our study. Although they are important at the end of life, evaluating these outcomes was not the main objective of our study. In addition, the effect of oxygen on dyspnea in patients with advanced cancer and low oxygen saturation has already been evaluated in randomized controlled trials [12–15]. Finally, the generalizability of the findings may be limited given that this study included patients with advanced cancer in home care with relatively short survival. The results of this study may not apply to patients with longer life expectancy.

## Conclusions

Oxygen use was not significantly associated with increased survival in patients with advanced cancer and low oxygen saturation, after adjusting for known prognostic factors and cardiopulmonary comorbidity. It may not be necessary to use oxygen for survival-prolongation in such patients, particularly in those without dyspnea.

## Data Availability

The datasets used and/or analyzed during the current study are available from the corresponding author on reasonable request.

## Abbreviations

CI: Confidence interval
ECOG: Eastern Cooperative Oncology Group
HR: Hazard ratio
ICPC-2: International Classification of Primary Care, second edition
IQR: Interquartile range
MICE: Multiple imputation by chained equations
PCU: Palliative care unit
SD: Standard deviation
